# Perception and Counseling for Cardiac Health in Breast Cancer Survivors Using the Health Belief Model: Qualitative Analysis

**DOI:** 10.2196/71062

**Published:** 2025-07-03

**Authors:** Sarah Tucker Marrison, Nicholas Shungu, Vanessa Diaz

**Affiliations:** 1 Medical University of South Carolina, 135 Cannon Street, Suite 405, Charleston, SC, United States, 1 8438761210

**Keywords:** cardiovascular health, cancer survivorship, lifestyle counseling, breast cancer, cancer survivors

## Abstract

**Background:**

Breast cancer survivors have increased cardiovascular risk compared to those without cancer history. Cardiovascular disease is the most common cause of death in breast cancer survivors. Cardiovascular risk in breast cancer survivors is impacted by both cancer treatment–associated effects and in risk factors for breast cancer and cardiovascular disease overlap. Strategies to improve screening for and management of cardiovascular disease in breast cancer survivors are needed to improve the delivery of survivorship care.

**Objective:**

This study aims to assess current cardiovascular risk counseling practices and perceived cardiovascular risk in breast cancer survivors.

**Methods:**

Semistructured interviews were conducted from May to December 2021 with breast cancer survivors identified as having a primary care clinician within an academic family medicine center in Charleston, South Carolina. The interview guide and content were developed using the Health Belief Model with a focus on cardiovascular risk behaviors, risk perception, and barriers to risk reduction. Analysis of categorical data was conducted by frequency and quantitative variables by mean and SD. Template analysis was performed for qualitative analysis. Outcome measures included self-reported history of cardiovascular disease, risk perception, and risk behaviors.

**Results:**

The average age of participants (n=19) was 54 (SD 7) years; 68% (13/19) were White and 32% (6/19) were Black or African American. Of the interviewed women, 90% (17/19) reported a personal history and 90% (17/19) reported a family history of cardiovascular disease. Only 53% (10/19) had previously reported receipt of cardiovascular counseling. Primary care most commonly provided counseling, followed by oncology. Among breast cancer survivors, 32% (6/19) reported being at increased cardiovascular risk, and 47% (9/19) were unsure of their relative cardiovascular risk. Factors affecting perceived cardiovascular risk included family history, cancer treatments, cardiovascular diagnoses, and lifestyle factors. Video (15/19, 79%) and SMS text messaging (13/19, 68%) were the most highly reported mechanisms through which breast cancer survivors requested to receive additional information and counseling on cardiovascular risk and risk reduction. Commonly reported barriers to risk reduction such as physical activity included time for meal planning and exercise, resources to support dietary and exercise changes, physical limitations, and competing responsibilities. Barriers specific to survivorship status included concerns for immune status during the COVID-19 pandemic, physical limitations associated with cancer treatment, and psychosocial aspects of cancer survivorship.

**Conclusions:**

Breast cancer survivors identified that factors associated with their cancer diagnosis and treatment both impacted their cardiovascular risk and introduced additional barriers to risk reduction. Potential strategies to improve counseling and awareness around cardiovascular risk include video and messaging platforms. Further risk reduction strategies should consider the unique challenges of cancer survivorship in delivery and implementation.

## Introduction

Breast cancer survivors are at increased risk of cardiovascular disease in part due to pre-existing comorbidities and cardiotoxicity associated with treatment [1]. Breast cancer survivorship has increased significantly, with over 3 million current breast cancer survivors. Breast cancer survivors are at increased risk of cardiovascular disease including heart failure, myocardial infarction, coronary artery disease, and vascular disease compared to those without a history of malignancy [2]. The etiology of the increased risk includes common risk factors, pre-existing comorbidities, and cardiotoxicity associated with breast cancer treatment. The likelihood of morbidity and mortality from cardiovascular disease exceeds that of breast cancer for many older women and those who have a personal history of cardiovascular disease [3-5]. In breast cancer survivors older than 50 years of age, 35% of nonbreast cancer–associated mortality was attributed to cardiovascular disease [6]. In one cross-sectional study, over 60% of breast cancer survivors were diagnosed with hypertension, over 50% with hyperlipidemia, and 5%‐6% had a reported history of heart failure or stroke [7]. Cardiovascular comorbidity is common in breast cancer with significant implications for morbidity and mortality requiring screening, counseling, and management with a multidisciplinary health care team including primary care.

Comorbid risk factors for breast cancer include increasing age, alcohol consumption, dietary patterns, family history of cardiovascular disease, elevated BMI, and physical inactivity [2]. Breast cancer treatments can increase the risk of cardiovascular disease, including chemotherapy, endocrine therapy, and radiation therapy, although the specific cardiovascular risk profiles vary based on treatment history [2,8]. Management of comorbid medical conditions has also been shown to be suboptimal during active cancer treatment. Cancer disease processes themselves may cause subclinical myocardial damage [9]. Current strategies for risk factor reduction in breast cancer survivors include aggressive management of comorbid conditions, including hypertension, diabetes, and hyperlipidemia, and promotion of lifestyle factors such as smoking cessation, maintaining an appropriate body weight, and increased physical activity [1,10-12]. Survivorship guidelines for breast cancer recommend screening and management of cardiovascular risk among patients with breast cancer; however, current screening practices and management are inadequate [3,13]. Lifestyle interventions including nutrition, physical activity, and weight management are essential components to integrate into survivorship care to reduce cardiovascular risk [14].

When considering cardiovascular disease and breast cancer risk, it is advised to weigh factors both of breast cancer reoccurrence and cardiovascular disease risk [15]. However, previous studies have identified that women at increased risk of cardiovascular disease also have an increased risk of cancer reoccurrence [16]. Interventions targeting cardiovascular risk reduction also have the potential to impact breast cancer outcomes. Despite this, limited qualitative and interventional studies have evaluated strategies and interventions for cardiovascular risk reduction in breast cancer survivors. Observational studies have demonstrated that increasing physical activity and dietary changes improve all-cause survival and improve cardiovascular risk in breast cancer survivors [17,18]. Pilot studies using exercise-based interventions have been demonstrated to improve cardiovascular fitness in breast cancer survivors. A randomized trial by Lee et al [19] demonstrated an improved cardiovascular risk profile following participation in a 16-week exercise program [20]. Cardiovascular risk reduction consistent with guidelines and evidence-based interventions focuses on smoking cessation, increased physical activity, and dietary changes, although the specific dietary recommendations vary based on guidelines and interventions studied [21]. The purpose of this project is to identify multilevel barriers and facilitators to cardiovascular risk reduction among breast cancer survivors to best advise breast cancer survivors and to support the development of interventions focused on the unique demands of breast cancer survivors following treatment.

## Methods

Qualitative analysis using semistructured interviews informed by the Health Belief Model was conducted to evaluate cardiovascular counseling and perceived risk in breast cancer survivors.

### Inclusion Criteria

Inclusion criteria included female breast cancer survivors aged 40‐65 years without metastatic disease seen by primary care at the Medical University of South Carolina in Charleston. The clinic population at the time of study initiation was predominately White and approximately 30% Black or African American. A majority of clinic patients were insured with either public or commercial insurance. For the purposes of this study, survivorship was defined as women who had completed their primary treatment for breast cancer. However, ongoing maintenance therapy, including endocrine therapy, was not considered an exclusion criterion.

### Interviews

Participants were contacted using MyChart messaging within the electronic health record. Potentially eligible participants were identified through an electronic health record data inquiry based on the history of breast cancer and primary care appointments at the academic medical center within the last 3 years. Only individuals who had opted into a system-wide query about research contact were included in outreach. Of the individuals contacted, 11% (19/170) participated in the study. Outreach was stratified by age (<50 years old and >50 years old) and race (White, Black, or African American) with outreach equally to these groups to promote representation consistent with clinic demographics. A total of 19 semistructured interviews were conducted on Teams (Microsoft Corp) using audio recording only by a trained female, master-level study coordinator. The interview duration ranged from 11 to 17 minutes. Interviews were conducted until saturation of depth and diversity of themes occurred. Neither the interviewer nor the study team had previous interactions with the participants. Study objectives were provided to interview participants prior to informed consent. Interviews were transcribed and reviewed for accuracy. Participants were not contacted following the initial interview. Participants were provided with contact information for the study team with an opportunity to reach out about any additional questions or study results. Our research team is composed of primary care clinicians and public health researchers involved in primary care research, including researchers with expertise in onco-primary care. Our team values patient self-efficacy and the importance of interdisciplinary coordination of care guided by a patient-centered approach to care. To reduce potential bias, the study team member completing the interviews was not involved in data coding. Coding was completed independently and reviewed by consensus.

### Conceptual Framework

The study was grounded in the Health Belief Model as a conceptual framework [22]. The Health Belief Model was selected based on its capacity to understand how individuals respond to communication of health-related information and their subsequent engagement in health-related behaviors. The Health Belief Model has been extensively used to assess cardiovascular risk perception, barriers to behavior change, and the development of interventions to address these [23-27]. More specifically, in this study, the focus on communication of information about cardiovascular risk in breast cancer survivors was predicted to inform and desire to and engagement in health behaviors to reduce cardiovascular risk. The interview guide was developed by the primary author after a review of previous literature, with revision following feedback from the research study team. Training was provided in the interview guide. Key constructs evaluated included perceived susceptibility to cardiovascular disease, perceived severity, self-efficacy, perceived barriers, and cues to action for engaging in cardiovascular risk reduction behaviors. Perceived susceptibility provides essential information on the participant’s assessment of the probability of cardiovascular disease. Perceived severity focused on the understanding of the potential severity of health impacts of cardiovascular disease and its relation to breast cancer survivorship status. Participants identified their perceived barriers to engaging in health behaviors to promote improved cardiovascular health. Self-efficacy was assessed based on the patient’s reported belief in their capacity to engage in health behaviors to reduce cardiovascular risk. Cues to action included experiences or actions within the participant’s environment that prompted the decision to engage in cardiovascular risk reduction behaviors. Interview questions also included questions about adherence to cancer survivorship guidelines.

### Template Analysis

Template analysis was conducted using an interview guide and codebook developed using the Health Belief Model [22,28-31]. Template analysis uses a structured template while still permitting a flexible coding process for the analysis of qualitative data. Since the interview guides in this study were based on the Health Belief Model framework, template analysis allowed researchers to use an initial template developed based on the constructs of the Health Belief Model framework, while also allowing codes to be updated in the coding process [30]. Coding was conducted by 3 independent coders with previous experience with qualitative analysis who each independently coded all transcripts. Following initial limited interview coding, further coding continued by consensus of themes which remained consistent across all coders. Nvivo (Lumivero) was used to assist in coding. Saturation of themes was reached after 19 interviews.

### Ethical Considerations

The study was deemed by the Medical University of South Carolina institutional review board as exempt (Pro00073820) with a waiver for written informed consent. All participants provided verbal consent for participation. Participants self-reported current exercise participation, consumption of fruits and vegetables, and current cardiovascular health conditions. Interview transcripts were deidentified prior to coding and analysis. Interview participants received a US $35 gift card as compensation for their time for participation.

### Statistical Analysis

Descriptive statistics including mean and SD were calculated for continuous variables and frequency data for qualitative variables.

## Results

### Participants

A total of 19 participants were enrolled in and completed interviews. The average participant age was 54 (SD 7; range 43‐66) years. Participants averaged 3 primary care and 3 oncology visits annually. Over half (10/19, 52%) of participants reported receiving counseling on cardiovascular risk. Counseling was provided by primary care (8/10, 80%) and oncology (3/10, 30%). Demographic information is provided in Table 1. Themes and illustrative quotes identified are displayed in Table 2. Themes were consistent across participants based on age and race.

**Table 1.** Demographic information of participants (n=19).

Characteristics	Participants, n (%)
Race	
Black	6 (31)
White	13 (68)
Education	
High school or less	2 (10)
Some college	5 (26)
Bachelor’s program	7 (37)
Master’s program or graduate school	5 (26)
Employment	
Full time	13 (68)
Part time	2 (11)
No current employment	4 (21)
Marital status	
Married	11 (58)
Not currently married	8 (42)
Treatment history	
Surgery	19 (100)
Chemotherapy	12 (63)
Radiation	14 (74)
Endocrine therapy	14 (74)
General health (participant report)	
Poor	2 (11)
Fair or good	12 (63)
Very good or excellent	5 (26)
Adherence to survivorship guidelines	
>150 minutes exercise weekly	6 (32)
>5 servings fruits or vegetables daily	3 (16)
History of personal cardiovascular disease	17 (90)
Current cardiac medications	10 (50)
Family history of cardiovascular disease	17 (90)

**Table 2.** Health Belief Model themes.

	Quotation example	Theme
Perceived barriers	“I’m almost 100 percent sure [my exercise limitations are] due to breast cancer treatment because I have some lung involvement. And so, you know, I’ve had issues with having a pleural effusion where it builds up the liquid in your lung.” [INT7]	General barriers identified included fatigue, limited time, and medications Treatment-associated barriers included physical limitations and associated anxiety and stress
Overcoming barriers	“But when I’m on the road, it’s hard to find foods that fall in those categories ... When I know ahead of time that I’m going to have a pick up at a certain time. I’ll make sure I either eat before I leave or I pack a lunch. But lots of times ... I only got like a 30 minute window to get them to pick it up.” [INT17] “So I think making the time about prioritizing ... prioritizing the time to exercise, and to shop appropriately, to eat healthy, so making the right decisions.” [INT2] “I think the main thing affecting me was I was kind of feeling because of what I went through, I was suffering a lot with PTSD and anxiety and all that. And I think because of that, I wasn’t focusing too much on my health. I was stress eating and gained 20 pounds because I was like so stressed out from what happened and being on the hormonal meds and shot every month, it just really messed with my I guess, my whole, like, energy level and motivation.” [INT4]	Discussions with clinicians identified as factors promoting change Scheduling considerations impact exercise and nutrition and mechanisms to prioritize change Individual motivation important determinant for cardiovascular risk reduction
Perceived susceptibility	“Genetics. On my father’s side, everyone has died of heart disease, and I apparently got his genes.” [INT3] “I would say it’s pretty high and I say that basically because when I had my radiation, they told me my tumor happened to be on my left side, so my radiation happened to be my heart. And so they told me that I was a high risk for congestive heart failure in the future.” [INT12] “I’ve never correlated the breast cancer to heart disease. I’m gonna need to think a little bit more about that. But I’ve never done that. So I would say no, because I just never thought about it in that way.” [INT2]	Individual factors impacting perceived risk include current personal cardiovascular disease, family history of cardiovascular disease, and lifestyle factors Cancer and treatment-associated factors impact perceived risk of breast cancer in some individuals although there was uncertainty in others
Communication regarding cardiovascular risk and cancer	“Possibly in the beginning ... My oncologist may have [discussed cardiovascular risk], but at that point, I think I was so overwhelmed with everything else that I probably didn’t listen that well.” [INT6] “So, like, if I am at risk as being a breast cancer patient for heart issues, then that’s something that I would want to stay on top of and have more information and have my primary care doctor or my oncologist talk to me about in ways that I could get on top of that before anything crazy happens.” [INT7]	Timing important factor in communication between health care team and patients about cardiovascular risk Communication limited about cardiovascular risk and breast cancer.
Self-efficacy	“I stopped smoking was one [way I took control of my health]. Because the reality of possibly having cancer in another location or not healing from the procedure. I never realized that they actually kept you from healing. And diet improved. Everything had to improve.” [INT5] “I’m a survivor, thank God. And I think it has a lot to do with my healthy choices. But if you don’t, if you’re not educated on that stuff, most people don’t know, you know. So it’s important to talk to professionals and see what’s the best thing for you to do.” [INT8]	Motivation to change and resources to support effective behavior change both reported as necessary by participants
Cues to action	“You know, there’s a publication that the [health system] puts out. I want to say four times a year ... There’s a lot of just good little health tips, reminders that just kind of keep things at the forefront when you’re starting to get busy.” [INT1]	Weight change and perception of body image motivation for lifestyle change New diagnoses of cardiovascular disease in an individual or relative impacted motivation to seek care Health promotion materials influence motivation for cardiovascular risk reduction

### Perceived Barriers

Perceived barriers included both factors unrelated to cancer, as well as those associated with the late and long-term effects of breast cancer and treatment. Commonly reported barriers included fatigue, time limitations to participation in exercise or meal planning, motivation to change, and available resources to support behavioral changes to support cardiovascular risk reduction. Cancer-specific barriers included physical limitations, including those from previous cancer treatments that impacted the capacity for exercise. Anxiety and stress, especially associated with changes in health status associated secondary to cancer and cancer treatment, were reported and identified as a barrier to behavior changes in exercise and nutrition. Medication side effects following treatment were cited by one participant as a limitation to participation in exercise.

### Overcoming Barriers

Engagement of the health care team and individual motivation for change were identified as factors promoting cardiovascular risk reduction behaviors. Participants reported that treatment of mental health conditions improved their capacity for behavior change. Participants highlighted the need for time management for meal preparation and exercise. The availability of group classes and discussions with clinicians in the health care team increased motivation for behavioral change.

### Perceived Susceptibility

Participants identified breast cancer treatment–associated factors including radiation and chemotherapy as contributing to cardiovascular risk. Others expressed uncertainty about the potential impact of cancer treatment on their cardiovascular risk but desired more information on this from their health care team. Individual factors associated with risk perception included current diagnosis of cardiovascular disease or history of a cardiovascular event. A family history of cardiovascular disease was reported as increasing an individual’s perceived personal risk of cardiovascular disease. Perceived protective factors included participant engagement in diet modification, regular exercise, and medication management of cardiovascular conditions.

### Communication Regarding Cardiovascular Risk and Cancer

Participants reported limited communication regarding cardiovascular risk specifically related to their cancer history. However, they desired to receive information on cardiovascular risk. Timing of communication about cardiovascular risk further removed from the time of initial diagnosis and treatment was preferred.

### Self-Efficacy

Participants identified self-efficacy as important in facilitating care and risk reduction. The ability to cook and resources for healthy food were identified as factors to support cardiovascular health. Motivation for change was identified as a necessary prerequisite for change.

### Cues to Action

Participants reported inciting stimuli to engage in risk reduction behaviors as a new diagnosis of cardiovascular health conditions either in themselves or in family members or friends. Individuals who were experiencing symptoms reported a stronger motivation to seek medical care and engage in risk-reduction behaviors. Participants valued outreach from trusted organizations, including health care organizations, about behaviors to improve health and reduce cardiovascular risk as important in keeping their desire for change at the forefront of their priorities.

### Sources of Information

The majority of individuals valued information from their health care team or other medical resources designed for patients. Specific sources of information on cardiovascular risk included clinicians, medical publications, and both medical and nonmedical internet sites. The timing of communication impacted the reception of the discussion regarding risk. Some participants reported the initial stages of treatment as overwhelming in the amount of information delivered and reported being more treatment-focused at that time. Participants did desire to receive information about their cardiovascular risk both from their oncologists, as well as their primary care clinician. Desired mechanisms of delivery of information on cardiovascular risk in survivors of breast cancer included video (n=15, 79%), SMS text messaging (n=13, 68%), telephone (n=11, 58%), and mobile apps (n=10, 53%).

### Summary

Based on the use of the Health Behavior Model, themes that promoted engagement in cardiovascular risk reduction were identified in each of the constructs assessed. Perceived barriers included both traditional barriers to cardiovascular risk reduction, as well as treatment-associated barriers derived from both mental and physical health. Factors promoting overcoming these barriers included clinician engagement with patients about cardiovascular risk reduction and the need for individual motivation for change to accomplish this. Although clinician communication was essential in promoting change, the timing of communication was identified as important. Immediately after diagnosis, breast cancer survivors preferred to focus discussion on treatment options. The availability of both financial resources and accessible resources supported the self-efficacy of participants with video and SMS text messaging as the preferred mediums for health information. Cues to action included changes in family and individual health. These interviews and themes have the capacity to inform further counseling and the development of interventions to support cardiovascular risk reduction both in breast cancer survivors and other cancer survivor populations.

## Discussion

### Principal Findings

Breast cancer survivors face additional cardiovascular risk following treatment, which impacts both overall and breast cancer–specific survival. Survivorship guidelines recommend screening and management strategies to reduce cardiovascular risk. Limited information is available on the barriers and facilitators of engaging breast cancer survivors in cardiovascular risk reduction behaviors. This qualitative study using semistructured interviews sought to evaluate facilitators and barriers to cardiovascular risk reduction in breast cancer survivors. Consistent with previous literature, uptake of survivorship guidelines including participating in physical activity and regular consumption of fruits and vegetables was limited [4]. The participants in the study overall received multiple treatment modalities that had the potential to increase individual cardiovascular risk.

The primary findings of the study included that there were opportunities to improve communication about cardiovascular risk in breast cancer survivors both within primary care and oncology. Breast cancer survivors valued communication about cardiovascular risk reduction, while also identifying the timing of delivering this information as important in engagement with subsequent risk reduction behaviors. Specifically, participants noted the importance of cardiovascular risk counseling as they progressed through treatment and into survivorship, rather than just at diagnosis. Many participants reported that they did not receive information on cardiovascular risk and were interested in learning more about their individual risks and the impact of their treatment on cardiovascular risk. These findings are in alignment with previous studies on both cancer survivors and health care professionals who identified that there was a knowledge gap among breast cancer survivors about cardiovascular risk. Cancer survivors consistently identified interest in discussions about heart disease and risk factors [32-34]. Although health care providers are generally aware of the increased cardiovascular risk in cancer survivors, system-based barriers include a lack of training, competing demands, and time limitations that limit the capacity for counseling and screening [34]. Furthermore, patient-level factors including socioeconomic factors and having a fatalistic outlook have been reported by health care clinicians as barriers to adopting risk reduction strategies [32,33]. In this study, the need for resources to promote self-efficacy was similarly identified by multiple participants even in a population with access to primary care [35].

Participants identified previously known barriers and facilitators to cardiovascular risk reduction, including motivation, time limitations, and resources to support efforts for change. Participants simultaneously identified breast cancer–specific factors as both significant components of their risk perception and as limitations to participation in risk-reducing behaviors. These same barriers were identified in previous formative research for intervention development, with the identification of cancer and other health conditions limiting the ability to increase physical activity. These studies simultaneously identified the need for individual motivation to facilitate change [34]. Participants in this study identified the need for multimodal changes, which aligns with previous literature supporting the greater efficacy of interventions that address factors beyond physical activity alone [30,31,34].

Breast cancer survivors desired additional communication about cancer-specific cardiovascular risk factors, preferably delivered after initial diagnosis and treatment have been established. Cardiovascular risk reduction strategies, including physical activity, have been demonstrated to reduce cardiovascular risk and improve quality of life, and therefore increased attention to cardiovascular risk reduction is essential to promoting improved survivorship care of breast cancer survivors [36,37]. Therefore, additional interventions that promote cardiovascular risk reduction and consider the elevated risks and unique barriers faced by breast cancer survivors are essential.

This study supports and aligns with existing literature regarding the need to support self-efficacy and consider the physical limitations of breast cancer survivors following treatment. Previous research identifies the critical role of clinicians in identifying and communicating the cumulative role of risk factors in cancer survivorship on cardiovascular risk and the importance of patient-clinician communication [38]. Engagement in communication about cardiovascular risk is limited and especially important in cancer survivors at increased risk, including those with existing cardiovascular disease and obesity. Primary care clinicians can serve an important role in screening for cardiovascular risk, promoting risk reduction, and pharmacologic management when indicated [39]. Based on this study, primary care clinicians often initiate this discussion and have an opportunity to improve communication about risk and promotion of health behaviors. When primary care clinicians were surveyed about barriers and enablers of care, system-level barriers including lack of time and training were critical to address to promote improved cardiovascular survivorship care [32]. Clinical risk assessment and management tools have been identified as strategies to improve the delivery of cardiovascular disease care to reduce morbidity and mortality. This study identifies the need to consider mental health support and treatment in facilitating cardiovascular risk reduction. In addition, it identifies the importance of timing in the delivery of cardiovascular risk-reducing education during the breast cancer treatment-survivorship continuum in order to optimize the uptake of risk-reducing measures.

### Strengths and Limitations

The proposed study is novel in its evaluation of breast cancer survivors–specific perceived risks and susceptibility to breast cancer, as well as potential survivorship-associated barriers to engagement in risk reduction. However, the study has a number of limitations. First, the study focuses on participants from a single academic medical center, which may limit its transferability. Further studies should include participants from a diversity of geographical locations and practice settings. A stratified recruitment approach was used to broaden the perspectives obtained as part of the interview process. Second, participants were selected based on having an existing primary care clinician, as the study included the role of primary care clinicians in survivorship. Future work would benefit from the inclusion of individuals with reduced access to care. Third, study findings may have been impacted by participation bias, where either the desire to engage in cardiovascular risk reduction behaviors or existing cardiovascular disease may have impacted the decision to participate. There are elevated rates of cardiovascular disease in breast cancer survivors, and this perspective is proportionally represented in this study. Another limitation is that the interview guide was not pretested before implementation. The interview guide was based strongly on the constructs of the Health Belief Model, which have been extensively studied. Future investigations could ensure the validity of the interview guide as related to capturing Health Belief Model constructs as related to cardiovascular risk in breast cancer survivors. Finally, the study is limited by the small sample size. This study focused on a limited number of individuals in order to obtain more breadth of information. While the achievement of theme saturation strengthens the study findings, future quantitative studies are needed to further evaluate the identified themes using a larger sample size to promote the development of interventions that improve communication between health care team members and breast cancer survivors about cardiovascular risk and engagement with risk reduction behaviors.

### Conclusions

Breast cancer survivors face unique cardiovascular risks influenced by their diagnosis and treatment, which introduce barriers to effective risk reduction. Participants in this study expressed a desire for enhanced counseling on cardiovascular health, highlighting a significant knowledge gap regarding their risks and the impact of cancer treatments. Many reported insufficient communication from health care providers about cardiovascular risks, particularly after the initial treatment phases. To address these issues, health care systems should leverage electronic communication methods, such as video and SMS text messaging, to deliver tailored cardiovascular risk information. Interventions must consider the specific challenges of survivorship, including physical limitations and mental health concerns, to effectively engage survivors in risk-reducing behaviors. This study highlights the importance of improving adherence to guideline-based care for breast cancer survivors and emphasizes the need for further research to develop comprehensive strategies that facilitate cardiovascular health in this population. By prioritizing timely and accessible information, we can enhance self-efficacy and support better health outcomes for breast cancer survivors.
